# [ ^18^F]PM‐PBB3‐PET Reveals Clinical and [ ^18^F]FDG‐PET Mimics of 4‐Repeat Tauopathy Caused by Creutzfeld‐Jakob Disease

**DOI:** 10.1002/mdc3.13683

**Published:** 2023-02-13

**Authors:** Nils Schröter, Ganna Blazhenets, Philipp T. Meyer, Michel Rijntjes, Joachim Brumberg

**Affiliations:** ^1^ Department of Neurology, Medical Center, Faculty of Medicine University of Freiburg Freiburg im Breisgau Germany; ^2^ Department of Nuclear Medicine, Medical Center, Faculty of Medicine University of Freiburg Freiburg im Breisgau Germany

**Keywords:** corticobasal syndrome, 4‐repeat‐tauopathy, Tau‐PET, Creutzfeld‐Jakob disease

This image depicts [^18^F] FDG and [^18^F]PM‐PBB3 uptake surface projections of two male patients with complex movement disturbances. They were referred for PET imaging with the suspected diagnosis of a 4‐repeat (4R)‐tauopathy Figure 1. Patient #1 (79 years) presented with an aggressive, non‐levodopa responsive hypokinetic‐rigid syndrome with early falls and a vertical supranuclear gaze palsy. Patient #2 (68 years) presented with unusually rapidly progressive dystonia, right‐sided rigor, bradykinesia, alien limb phenomenon and action myoclonus of the right arm, likewise without response to levodopa. Disease duration was 11 and 5 months, respectively. CSF levels of tau and amyloid in either patient were not indicative for Alzheimer's disease. In both, [^18^F] FDG‐PET revealed a corticobasal degeneration‐like bilateral reduction of cerebral glucose metabolism in frontoparietal cortical areas (arrowheads) and in the thalamus.^1^ A clear asymmetry to the detriment of the right hemisphere was observed in #1, whereas hypometabolism of the left hemisphere was only slightly pronounced in #2. Subsequent imaging with tau ligand [^18^F]PM‐PBB3^2^ showed elevated binding (arrowheads, asterisks indicate unspecific uptake of the choroid plexus) suggestive for pathological tau aggregates in frontal and parietal areas of #1, supporting the diagnosis of a 4R‐tauopathy.^2^ In contrast, the lack of specific [^18^F]PM‐PBB3 binding in patient #2 questioned the diagnosis of a 4R‐tau related corticobasal syndrome (CBS; eg, progressive supranuclear palsy—CBS). Diagnosis of Creutzfeld‐Jakob disease in this patient was made via 14–3‐3 and PrPSc aggregation assays. In conclusion, [^18^F]PM‐PBB3‐PET reveals clinical and [^18^F] FDG‐PET mimics of 4R‐tau CBS caused by Creutzfeld‐Jakob disease.

**Fig. 1:**
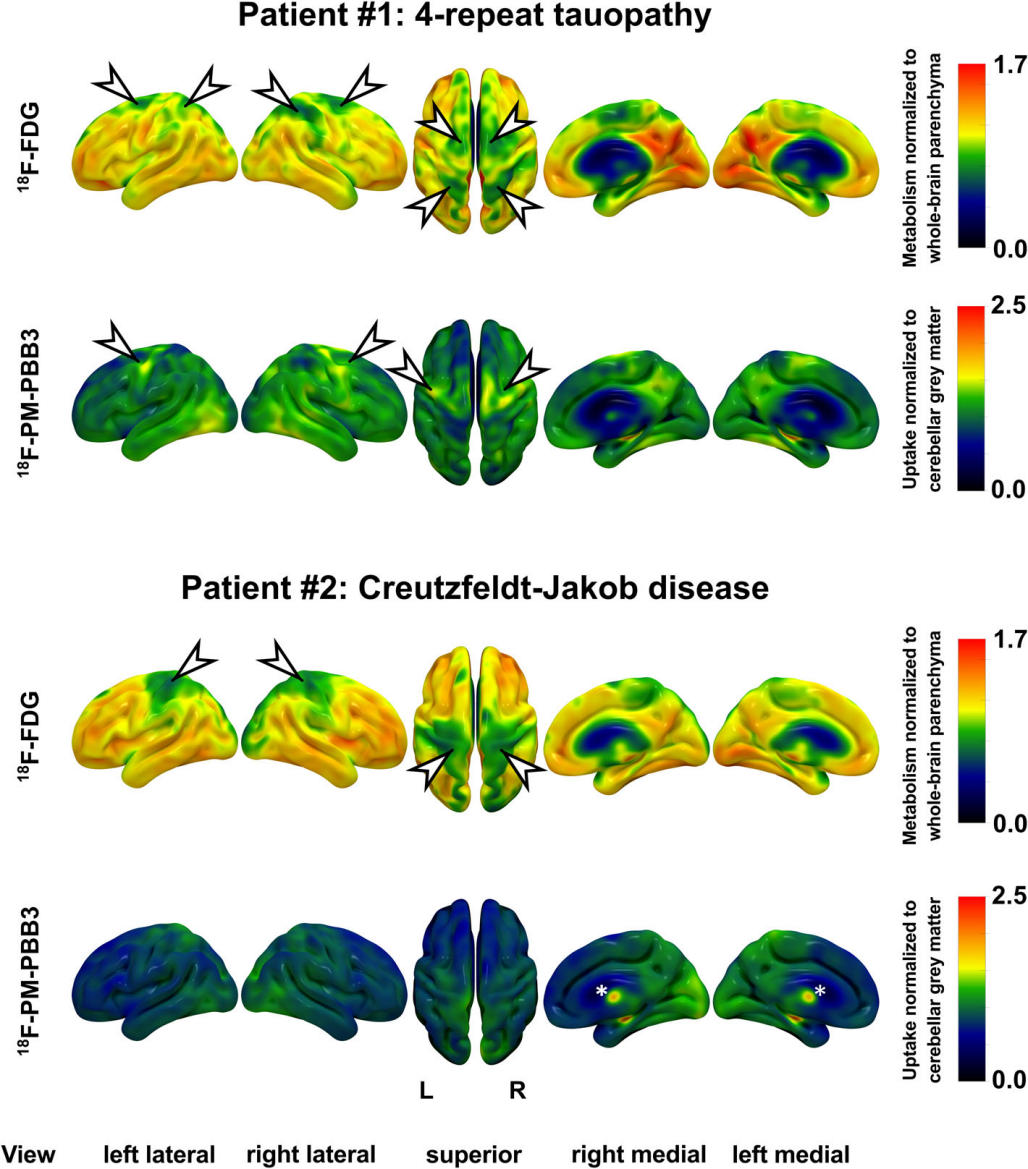
This image depicts [^18^F] FDG and [^18^F]PM‐PBB3 uptake surface projections of two male patients with complex movement disturbances. Colorbars on the right side indicate uptake ratios. L, left, R right.

## Author Roles

(1) Research project: A. Conception, B. Organization, C. Execution; (2) Statistical analysis: A. Design, B. Execution, C. Review and critique; (3) Manuscript: A. Writing of the first draft, B. Review and critique.

N.S.: 1A, B, 1C, 2C, 3A.

G.B.: 1C, 2B, 2C, 3B.

P.T.M.: 1B, 2A, 2C, 3B.

M.R.: 1B, 2C, 3B.

J.B.: 1C, 2A, 2C, 3A.

## Disclosures

**Ethical Compliance Statement:** The authors confirm that the approval of an institutional review board was not required for this work. Both patients gave written informed consent for the scientific use of imaging and clinical data.

**Funding Sources and Conflicts of Interest:** Nils Schröter, Berta‐Ottenstein‐Programme for Clinician Scientists, Faculty of Medicine, University of Freiburg. The authors have no potential conflict of interest to report. We confirm that we have read the Journal's position on issues involved in ethical publication and affirm that this work is consistent with those guidelines.

**Financial Disclosures for the Previous 12 Months:** The authors have nothing to disclose.

## References

[1] Niethammer M , Tang CC , Feigin A , et al. A disease‐specific metabolic brain network associated with corticobasal degeneration. Brain 2014;137:3036–3046. 25208922 10.1093/brain/awu256 PMC4208467

[2] Tagai K , Ono M , Kubota M , et al. High‐contrast In vivo imaging of tau pathologies in Alzheimer's and non‐Alzheimer's disease tauopathies. Neuron 2021;109(42–58):e48. 10.1016/j.neuron.2020.09.042 33125873

